# Liver fat in adult survivors of severe acute malnutrition

**DOI:** 10.1038/s41598-022-07749-5

**Published:** 2022-03-07

**Authors:** Debbie S. Thompson, Tamika Y. N. Royal-Thomas, Ingrid A. Tennant, Deanne P. Soares, Christopher D. Byrne, Terrence E. Forrester, Peter D. Gluckman, Michael S. Boyne

**Affiliations:** 1 Caribbean Institute for Health Research, The University of the West Indies, Kingston 7, Jamaica; 2 Department of Mathematics, The University of the West Indies, Kingston, Jamaica; 3 Department of, Surgery, Radiology, Anesthesia and Intensive Care, The University of the West Indies, Kingston, Jamaica; 4 Nutrition and Metabolism Unit, Faculty of Medicine, University of Southampton, Southampton, UK; 5 Institute of Developmental Sciences and NIHR Biomedical Research Centre, University of Southampton and University Hospital Southampton, Southampton, UK; 6 UWI Solutions for Developing Countries, The University of the West Indies, Kingston, Jamaica; 7 UK Centre for Human Evolution, Adaptation and Disease, Liggins Institute, University of Auckland, Auckland, New Zealand; 8 Department of Medicine, The University of the West Indies, Kingston, Jamaica

**Keywords:** Medical research, Risk factors

## Abstract

The association between severe acute malnutrition (SAM) in early childhood and liver fat in adults is unknown. We hypothesized that exposure to SAM, especially severe wasting, is associated with fatty liver later in life. In this observational study, abdominal CT was used to quantify mean liver attenuation (MLA) and liver:spleen attenuation ratio (L/S). Birth weight (BW), serum lipids, insulin resistance (homeostatic model assessment), anthropometry and intrabdominal fat were collected. Mean differences between diagnostic groups were tested and hierarchical regression analysis determined the best predictors of liver fat. We studied 88 adult SAM survivors and 84 community participants (CPs); age 29.0 ± 8.4 years, BMI 23.5 ± 5.0 kg/m^2^ (mean ± SDs). SAM survivors had less liver fat than CPs (using L/S) (*p* = 0.025). Severe wasting survivors (SWs) had lower BW (-0.51 kg; *p* = 0.02), were younger, thinner and had smaller waist circumference than oedematous malnutrition survivors (OMs). In the final regression model adjusting for age, sex, birth weight and SAM phenotype (i.e., oedematous malnutrition or severe wasting), SWs had more liver fat than OMs (using MLA) (B = 2.6 ± 1.3; *p* = 0.04) but similar liver fat using L/S (*p* = 0.07) and lower BW infants had less liver fat (MLA) (B = -1.8 ± 0.8; *p* = 0.03). Greater liver fat in SWs than OMs, despite having less body fat, supports our hypothesis of greater cardiometabolic risk in SWs. Other postnatal factors might influence greater liver fat in survivors of severe wasting, suggesting the need to monitor infants exposed to SAM beyond the acute episode.

## Introduction

Severe acute malnutrition (SAM) in children less than 5 years remains a significant global problem, and malnutrition contributes to 45% of all child deaths worldwide^1^. As more children survive the episode, the long-term effects of SAM and its possible associations with chronic non-communicable diseases (NCDs) become increasingly relevant. Fatty liver disease (FLD) represents a spectrum of disorders, spanning from simple fatty infiltration to an intermediate, inflammatory stage (non-alcoholic steatohepatitis) to an irreversible end stage (cirrhosis). Despite a benign clinical presentation, FLD is a significant public health concern and the most significant hepatic disorder in high-resource settings^2^. It is also considered an independent risk factor for cardiovascular disease^3^ and has established associations with obesity^4^ and insulin resistance^5^.

Intrauterine growth retardation has been previously associated with later fatty liver disease^6^, but the association between SAM in early childhood and adult fatty liver is unknown. The available data on this topic which comes from the 1960–1970s show that liver fat accumulation has been observed in both SAM phenotypes (defined by oedematous malnutrition or severe wasting) during the acute episode^7^, but this appears to resolve completely during childhood^8^. Women who had fetal and childhood-exposure to the 1959–1961 Chinese Famine had a significantly higher prevalence of moderate-to-severe hepatic steatosis than women who were not exposed, but they also had higher prevalence of obesity as adults^9^ which was not adjusted for in the analyses. Similarly, individuals from the Helsinki Birth Cohort who were small during early childhood had a higher risk of developing FLD, but this risk was higher in those who were obese as adults^10^. It is therefore unclear whether there is an association between severe acute malnutrition in early life and fatty liver in adults that is independent of adult overweight/obesity.

Furthermore, as infants with the severe wasting phenotype of SAM weigh less at birth than those with oedematous malnutrition^11^, these SAM phenotypes may themselves impute different degrees of subsequent cardiometabolic risk. As evidence, we have previously reported worse beta cell function, greater glucose intolerance and a tendency towards greater insulin resistance among adult survivors of severe wasting compared to survivors of oedematous malnutrition^12^.

This study aimed to measure and compare liver fat in adult SAM survivors and matched community participants as well as to compare liver fat in adult survivors of severe wasting and oedematous malnutrition. We hypothesized that adults who were exposed to severe malnutrition in early life would have more liver fat than participants from their communities who were not previously exposed to SAM. We further hypothesized that adults who were survivors of severe wasting have more liver fat than adult survivors of oedematous malnutrition. These hypotheses were tested in a unique cohort of Afro-Caribbean adult survivors of severe acute malnutrition and community participants.

## Methods

### Study design/subjects

A retrospective cohort was assembled by reviewing ward records of patients admitted to the Tropical Metabolism Research Unit of the University of the West Indies between 1963 and 1993. Using the Wellcome criteria, patients were classified as having severe wasting (weight-for-age < 60%) or oedematous malnutrition (60–80% weight-for-age, plus the presence of oedema) in comparison to the National Centre for Health Statistics (NCHS) standard growth curves^13^. Of 1336 patients admitted during the period, 729 individuals were traced, 316 had basic anthropometric measurements^11^ and 92 underwent abdominal CT scans (Supplementary Fig. 1). We recruited 87 community participants that had never been hospitalized for SAM; they were matched based on age ± 5 years, sex, and BMI ± 2 kg/m^2^ and were drawn from the same communities of the SAM cases. Persons with sickle cell haemoglobinopathy, a history of liver disease or taking medications that cause hepatic abnormalities were excluded from the study and participants with a self-reported alcohol intake of more than 14 drinks per week (males) and more than 7 drinks per week (females)^14^ were excluded from the analyses.

The Faculty of Medical Sciences/University Hospital of the West Indies Ethics Committee approved the study protocol (ECP17: 14/15) and each participant gave written informed consent. The procedures followed were in accordance with the ethical standards of the University of the West Indies and the Ministry of Health and Wellness.

### Measurements

After a 10 h overnight fast, participants reported to the Tropical Metabolism Research Unit where they completed a staff-administered health questionnaire. Birth weights documented during admission to TMRU ward as children were abstracted from patient records, and objective data sources were utilized to minimise recall bias. No birth weight data was available for community participants.

#### Anthropometry and body composition

Body weight was measured to the nearest 0.1 kg and height and waist circumference to the nearest 0.1 cm using a standardized protocol^15^. A whole-body DEXA scan was performed on each participant to measure body composition (Lunar Prodigy, GE Health Care, USA).

#### Assays

10 mLs of blood was taken through antecubital venipuncture for fasting glucose, insulin, lipids and alanine aminotransferase (ALT) measurements. Glucose concentration was determined by the glucose oxidase method on an autoanalyzer. Insulin concentration was measured with an ELISA assay (ALPCO Diagnostics, Salem, NH, USA)^16^, which had analytical sensitivity of 0.399 μIU/ml and an intra-assay coefficient of variation < 5% in our laboratory. Total cholesterol, HDL-cholesterol, triglycerides and ALT were measured by enzymatic techniques using a COBAS INTEGRA 400 Plus Analyzer (Roche Diagnostics, IN, USA). LDL-cholesterol was calculated by the Friedewald formula^17^.

#### Calculations

Homeostatic model assessment-insulin resistance (HOMA-IR) = I_0 _× G_0_/22.5, where G_0_ and I_0_ reflect basal (fasting) glucose and insulin in SI units^18^.

#### Liver and visceral fat

Abdominal CT scans (Phillips Brilliance 64-slice scanner) were undertaken to measure hepatic steatosis and visceral adiposity. A calibration control (phantom) was placed between the table and the participant’s back during the CT scan and this was used to standardize all liver measurements. With the participant in a supine position, a single 5 mm slice (120 kVp, 100 mA) was taken at the T12/L1 intervertebral disc space to include both the liver and the spleen. The scan was not contrast-enhanced. A second scan was located at the L4/L5 disc space to measure total abdominal and visceral fat area, mass and volume.

### Data analysis

eFilm Workstation 3.1 (Merge Healthcare, Chicago, IL, USA) was used to interpret the images from the abdominal CT scan and quantify liver fat. Three regions of interest (ROIs), each measuring a minimum 1 cm^2^ were consistently placed in the image of the liver as follows: right posterior lobe, right anterior lobe and left lobe. Similarly, one ROI was placed on the image of the spleen. Each ROI had a reading for mean attenuation in Hounsfield units (HU) ± standard deviation which were used to calculate mean liver attenuation (MLA) as well as the attenuation of the liver compared to that of the spleen; the liver: spleen ratio (L/S). MLA of < 40 HU and LS ratio of < 1 denote hepatic steatosis > 30% (moderate-severe fatty liver) and both MLA and L/S have an inverse relationship with liver fat^19,20^.

Images taken from the CT scanner were transferred to the Tissue Composition Module Beta 1.0 software package (Mindways, Austin, TX, USA); https://www.qct.com/TCM.html^21^, for estimation of total and intra-abdominal fat area, volume and mass using the QCT Pro software 2007, Version 4.2.3; (Mindways, Austin, TX), https://www.qct.com/QCTPro.html^22^. On each CT image, total adipose area (TAA) and visceral adipose tissue (VAT) were measured, and subcutaneous adipose tissue (SAT) was calculated (TAA-VAT).

### Statistical analysis

Assuming a power of 80%, an alpha of 0.05, a minimal clinically relevant difference of 5 Hounsfield units and a standard deviation of 11.5^14^, a sample size of 83 subjects per group was needed to answer the research question. A total of 179 participants underwent abdominal computed tomography. Student’s t-tests (adjusted for unequal variances) were used to test mean differences in normally distributed continuous variables between diagnostic groups i.e., severe wasting survivors (SWs) vs oedematous malnutrition survivors (OMs), SAM survivors vs community participants. Categorical variables were compared using the chi-squared test. Comparison of means among all three diagnostic categories (SWs, OMs, community participants) was undertaken using one-way ANOVA and the Mann–Whitney test where data were skewed. The final analysis of the data was based on the model that best identified liver fat using hierarchical multiple regression analysis. Although MLA was chosen as main estimate of liver fat, L/S is also used in the analyses. Both outcome measures of liver fat (MLA and L/S) were analyzed as continuous variables and listwise deletion was used to handle missing data in the regression models. Predictor variables were SAM exposure and SAM phenotype (i.e., severe wasting or oedematous malnutrition). Potential confounders were age, sex, birth weight and adult BMI and insulin resistance. Stata version 12.1 and SPSS 19.0 were used for all statistical analyses. Two-sided *p*-values were reported and a *p*-value ≤ 0.05 were considered statistically significant.

## Results

Data for 172 participants (88 adult SAM survivors and 84 community participants) were analyzed after 7 participants were excluded; 5 as they had no liver image on CT scan and 2 as their CT scans were delayed by more than 3 months after body composition was assessed by DEXA. As more participants had MLA (n = 169) than L/S (n = 133), MLA was selected as the main estimate of liver fat, however, data for L/S ratio was also analyzed as it is a recognized estimate of liver fat.

### Participant characteristics

53% of the 172 participants were male, age was 29.0 ± 8.4 years, BMI 23.5 ± 5.0 kg/m^2^ (means ± SDs). The 88 malnutrition survivors that had abdominal CT scans were similar in age, sex and BMI to the 228 other malnutrition survivors that did not undergo CT scans (*p* > 0.16). Mean liver attenuation was 63.5 ± 4.5 HU with a range of 53.4–73.9 HU. LS ratio was 1.2 ± 0.1 and the range was 0.9–1.58. Based on L/S, 2.9% of the participants had moderate-to-severe fatty liver and no participant met the criteria for moderate-to-severe fatty liver using MLA.

### SAM survivors versus community participants (CPs)

In addition to being of similar age, sex and BMI, SAM survivors and community participants had similar waist circumference and visceral adipose tissue. However, SAM survivors had higher serum HDL-C, lower serum LDL-C and higher L/S (i.e., less liver fat) than community participants (Table 1). Participant diagnosis (i.e., SAM survivor vs community participant) was associated with L/S (*p* = 0.029), but not MLA (*p* = 0.11) in an age and sex-adjusted regression model, i.e., SAM survivors had less liver fat (L/S). The ratio of visceral adipose tissue to subcutaneous adipose tissue ratio (VAT: SAT) was inversely associated with MLA (*p* = 0.028), but not L/S (*p* = 0.6). BMI was not significant when added to these models nor did BMI alter the reported associations.

**Table 1:** Clinical and metabolic characteristics of 88 Afro-Caribbean adult survivors of severe malnutrition (SOMs) and 84 community participants (CPs).

	Severe wasting survivors (SWs) (45)	Oedematous malnutrition survivors (OMs) (43)	*P-*value SWs vs OMs	Survivors of malnutrition (SOMs) (88)	Community participants (CPs) (84)	*P-*value SOMs v CPs
Age (years)	26.6 ± 8.8	30.7 ± 8.8	0.02	28.7 ± 8.8	28.8 ± 7.9	0.82
% Women	40	51	0.3	45	47	0.83
Birth weight (kg)	2.5 ± 0.8	3.0 ± 0.7	0.007	2.7 ± 0.8	–	–
BMI (kg/m^2^)	22.3 ± 5.4	25.1 ± 5.3	0.01	23.6 ± 5.5	23.4 ± 4.3	0.82
Waist (cm)	76.0 ± 12.9	81.7 ± 12.3	0.03	78.7 ± 12.8	78.4 ± 11.1	0.85
Fat mass (kg)	8.3 (3.9, 15.7)	16.0 (4.5, 31.0)	0.05	10.5 (4.1, 23.6)	11.2 (4.9, 21.9)	0.93
Lean Mass (kg)	46.6 (38.7, 53.9)	46.9 (40.0, 56.3)	0.21	46.7 (39.8, 54.6)	49.5 (38.2, 58.8)	0.27
% Body fat	16.1 (6.7, 29.2)	25.9 (7.6, 40.9)	0.07	18.1 (7.0, 35.1)	19.8 (7.6, 36.6)	0.94
VAT area (cm^2^)	27.3 (16.3, 45.0)	36.8 (19.4, 56.1)	0.68	35.3 (18.0, 47.9)	34.4 (16.3, 53.9)	0.66
SAT area (cm^2^)	66.5 (20.2, 130.0)	121.3 (30.9, 276.6)	0.09	86.4 (24.7, 219.3)	88.9 (22.3, 189.7)	0.41
VAT:SAT ratio	0.5 (0.2, 1.1)	0.3 (0.2, 0.7)	0.46	0.4 (0.2, 0.8)	0.4 (0.3, 0.7)	0.32
Mean Liver Attenuation (Hounsfield units)	63.7 ± 4.4	64.5 ± 4.9	0.45	64.1 ± 4.6	63.0 ± 4.2	0.11
L/S	1.2 (1.1, 1.3)	1.2 (1.2, 1.3)	0.71	1.2 (1.1, 1.3)	1.2 (1.1, 1.2)	0.025
Fasting Glucose (mmol/L)	4.6 ± 0.5	4.6 ± 0.4	0.66	4.6 ± 0.5	4.6 ± 0.5	0.69
Fasting Insulin (µIU/mL)	3.8 (2.5, 5.9)	3.3 (2.2, 5.5)	0.91	3.6 (2.4, 5.7)	3.2 (1.6, 7.5)	0.40
HOMA-IR	0.8 (0.5, 1.1)	0.7 (0.4, 1.1)	0.64	0.7 (0.5, 1.1)	0.7 (0.3, 1.4)	0.35
Total-C (mmol/L)	3.7 ± 0.8	3.8 ± 0.8	0.33	3.8 ± 0.8	4.0 ± 0.8	0.06
HDL-C (mmol/L)	1.3 ± 0.3	1.3 ± 0.3	0.29	1.3 ± 0.3	1.1 ± 0.3	0.0001
LDL-C (mmol/L)	2.1 ± 0.6	2.2 ± 0.7	0.72	2.1 ± 0.7	2.5 ± 0.9	0.0006
Triglyceride (mmol/L)	0.7 ± 0.2	0.8 ± 0.5	0.36	0.7 ± 0.4	0.7 ± 0.3	0.63
ALT (IU/L)	8.0 (6.0, 10.0)	8.0 (6.0, 12.3)	0.97	8.0 (6.0, 11.0)	8.0 (6.0, 10.0)	0.15

Characteristics of the study sample were compared by the independent sample t-test for continuous variables, and Pearson Chi square test for categorical variables. Normally distributed data are presented as means ± SDs and skewed data as medians (quartiles).

*VAT* visceral adipose tissue, *SAT* subcutaneous adipose tissue, *VAT:SAT ratio* VAT/SAT, *L/S* liver spleen ratio, *HOMA-IR* homeostatic model assessment of insulin resistance, *HDL-C* high-density lipoprotein cholesterol, *LDL-C* low-density lipoprotein cholesterol, *ALT* alanine aminotransferase.

### Severe wasting survivors (SWs) versus oedematous malnutrition survivors (OMs)

When the 88 SAM survivors were disaggregated by SAM phenotype (i.e., severe wasting and oedematous malnutrition), SWs had a lower birth weight (≈ 500 g) and were younger, with a lower BMI, waist circumference and fat mass than OMs (Table 1). SWs and OMs had similar visceral adipose tissue (VAT) and liver fat (MLA and L/S). When L/S was compared in the three diagnostic groups, survivors of oedematous malnutrition had 5.4% less liver fat than community participants (i.e., 1.26 vs. 1.19; 95% CI 0.1–11.1%), while survivors of severe wasting and community participants had similar amounts of liver fat.

**Figure 1:**
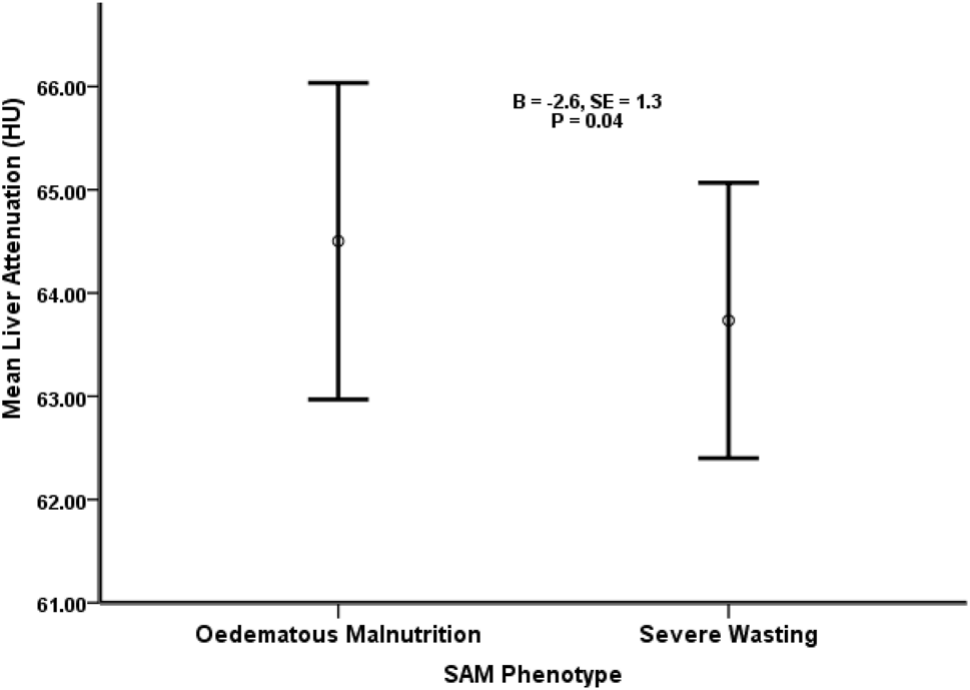
Regression of mean liver attenuation against SAM phenotype (adjusted for age, sex and birth weight) in 88 SAM survivors. The mean liver attenuation + SD as bars in Hounsfield units (HU) is shown for adult survivors of oedematous malnutrition and severe wasting.

To examine the relationship between SAM phenotype and liver fat, a hierarchical multiple regression analysis was performed using mean liver attenuation and L/S as dependent variables and with severe wasting as the reference diagnosis in the SAM phenotype variable. The results of each step in the regression analysis with mean liver attenuation as the dependent variable, regression coefficients and associated *P*-values are shown in Table 2. The analysis revealed that the model adjusting for age, sex, birth weight and SAM phenotype best predicted mean liver attenuation, having the greatest R^2^ change (0.079) and a significant F change (*P* = 0.028). This model explains 10% of the variability in mean liver attenuation (R^2^ = 0.100) (Table 2). In this model, survivors of severe wasting had more liver fat than survivors of oedematous malnutrition (2.61 ± 1.3, B ± SE) (*p* = 0.04) (Table 2, Fig. 1).

**Table 2:** Hierarchical multiple regression analysis of variables predicting mean liver attenuation in 88 adult survivors of severe acute malnutrition.

Models	Predictors	B	SE	*P*-value	95.0% CI for B		R^2^	R^2^ change	*P*-value F change
					Lower	Upper			
Step 1	Age	− 0.026	0.059	0.659	− 0.143	0.911	0.021		
	Sex	− 0.968	0.993	0.332	− 2.942	1.007			
	Oedematous malnutrition	0.957	1.014	0.343	− 1.050	2.981			
Step 2	Age	− 0.091	0.074	0.224	− 0.238	0.057	0.100	0.079	0.028*
	Sex	− 0.682	1.169	0.562	− 3.020	1.655			
	Oedematous malnutrition	2.614	1.272	0.044*	0.071	5.158			
	Birth weight (kg)	− 1.790	0.814	0.032*	− 3.417	− 0.163			
Step 3	Age	− 0.132	0.082	0.115	− 0.296	0.033	0.118	0.018	0.267
	Sex	− 1.276	1.282	0.323	− 3.840	1.287			
	Oedematous malnutrition	2.428	1.280	0.063	− 0.133	4.989			
	Birth weight	− 1.827	0.813	0.028*	− 3.452	− 0.201			
	BMI (kg/m^2^)	0.137	0.123	0.268	− 0.108	0.382			

*P < 0.05.

*B* unstandardized regression coefficient, *SE* standard error, *P* *p*-value for the unstandardized regression coefficient for the model, *CI* confidence intervals, *R*^*2*^ coefficient of determination, *R*^*2*^* change* the improvement in R-square when a predictor is added, *P-value F-change* p-value for the test of the R^2^ change. Coding for sex was male (0) and female (1). Coding for SAM diagnosis was oedematous malnutrition (1) and severe wasting (2). Mean liver attenuation has an inverse association with liver fat.

When HOMA was added to this model, survivors of severe wasting still had more liver fat than survivors of oedematous malnutrition (2.79 ± 1.4, B ± SE) (*p* = 0.04). However, when BMI was added to the model, severe wasting survivors showed only a tendency towards greater liver fat compared to kwashiorkor survivors (*p* = 0.063) (Table 2). Additionally, SAM phenotype was not associated with MLA after separately adjusting for mean waist circumference (*p* = 0.38), percent body fat (*p* = 0.2) and VAT: SAT (*p* = 0.46) (data not shown). SAM phenotype was not significant in a similar age, sex and birth weight-adjusted regression on L/S (*p* = 0.255).

**Figure 2:**
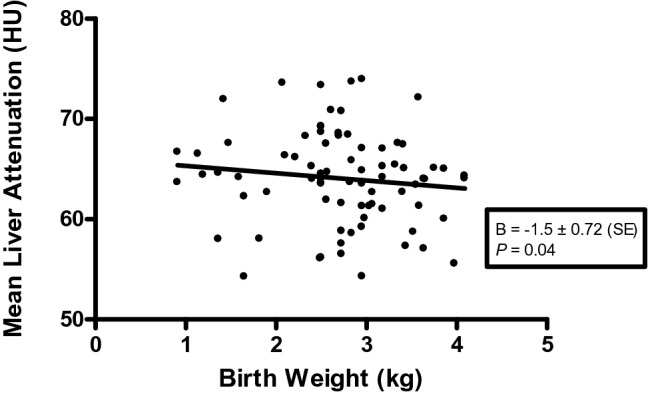
Regression of mean liver attenuation against birth weight (adjusted for age, sex and SAM phenotype) in 88 SAM survivors.

Birth weight had an inverse association with MLA in the final regression model (B = − 1.79, SE = 0.81; *p* = 0.03) (Table 2, Fig. 2) which was not affected when BMI was added to the model (Step 3) (*p* = 0.028). When L/S was the outcome variable, birth weight was not associated with L/S (B = -0.07, SE = 0.039; *p* = 0.066). BMI did not alter these reported associations. Of note, the birth weight/SAM phenotype interaction term was not significant.

## Discussion

Our data contribute to the literature on the effects of early life malnutrition on adult fatty liver as we describe long-term associations between birth weight and fatty liver in adulthood, as well as differences between adult survivors of the two phenotypes of severe acute malnutrition. In this sample of adult SAM survivors and participants from their communities, roughly 3% had moderate to severe fatty liver based on L/S. We demonstrated that survivors of severe wasting had more liver fat than survivors of oedematous malnutrition after adjusting for birth weight.

The relatively young age of the participants and their lean BMI suggest that their risk factors for fatty liver were relatively low. Additionally, they might not have been exposed to an environment that was sufficiently obesogenic to contribute to the development of moderate-to-severe fatty liver. Indeed, these participants might be more likely to have no (< 5% of liver weight) or mild (5–30% of liver weight) liver fat accumulation. Finally, given the reported lower prevalence of NAFLD in Blacks (11%) compared to Caucasians (15%) and Hispanics (28%)^23^, we cannot rule out the possible additional influence of ethnicity in this Afro-Caribbean group.

### Liver fat in adult SAM survivors compared to community participants

Despite being of similar age, sex and BMI, adult SAM survivors were observed to have less liver fat than community participants in both univariate and multivariate analyses. The difference in liver fat, however, is very small and of questionable clinical significance. Additionally, lower liver fat in SAM survivors appears to be driven by lower liver fat in survivors of oedematous malnutrition. While higher HDL-C and lower LDL-C in malnutrition survivors appears to suggest a more favourable cardiovascular risk profile in this group of SAM survivors compared to unexposed persons from their communities, it is possible that factors occurring after SAM exposure could exert a greater influence than the risk caused by exposure to malnutrition in early childhood. However, the fact that most of the adult SAM survivors continued to live in their native environment suggests they may have experienced continued exposure to a non-obesogenic environment.

### Liver fat in survivors of severe wasting and oedematous malnutrition is moderated by adult BMI

Survivors of severe wasting were younger, with lower BMI, lower fat mass and smaller waist circumference than survivors of oedematous malnutrition. Despite this, they had more liver fat than survivors of oedematous malnutrition in a model adjusting for birth weight. In this instance, birth weight has the effect of partially masking the association the association between SAM phenotype and liver fat. We posit that greater liver fat in adult survivors of severe wasting could also be influenced by childhood factors such as feeding and catch-up growth during and after nutritional rehabilitation.

It is notable that in this group of participants, greater liver fat in survivors of severe wasting was not influenced by insulin resistance (as estimated by HOMA-IR). While this finding might be surprising, we previously reported that in this population, HOMA-IR compared poorly with other measures of insulin sensitivity, specifically whole-body glucose disposal, the gold standard measure of insulin resistance^24^. Indeed, fasting indices of insulin resistance, such as HOMA-IR, have been shown to be less accurate in subjects with normal or near normal weight^25^.

Although the difference in liver fat is small, our data suggest that survivors of severe wasting, who had a lower birth weight and are presumably better adapted to reduced postnatal caloric intake, are at greater risk from an obesogenic lifestyle. Additionally, adult body size and adiposity (BMI, waist circumference, percent body fat and VAT: SAT) appear to influence the relationship between SAM phenotype and liver fat (MLA). The clear and novel implication is that survivors of severe wasting could be at greater risk of fatty liver if they become overweight or obese; a finding that has not been previously reported to our knowledge. This finding may indeed be consistent with the phenomenon of rapid catch-up growth in childhood negatively influencing later cardiovascular disease risk.

The specific finding of greater liver fat in adult survivors of severe wasting supports our hypothesis and is consistent with previous reports of greater cardio-metabolic risk in this cohort of malnutrition survivors i.e. higher post-challenge glucose levels, higher fasting hyperinsulinaemia, worse beta-cell function and a tendency towards more insulin resistance in survivors of severe wasting^12^. As the variables in the model accounted for very little of the variability in mean liver attenuation, it is likely that other prenatal (genetics, maternal BMI, maternal diet during pregnancy and gestational weight gain) and postnatal factors (catch-up growth during recovery from malnutrition and adult factors such as diet, obesity and body composition) could have a stronger influence on liver fat in these adults.

### Birth weight is associated with liver fat in SAM survivors

We demonstrated that, among malnutrition survivors, children with lower birth weight had less liver fat after adjusting for age, sex and SAM phenotype. This finding is inconsistent with the developmental origins literature, as low birth weight has been associated with paediatric FLD^26^ and exposure to the Great Famine in early life was shown to have a sex-specific association with moderate-to severe NAFLD in Chinese adults^27^. Our finding might mean that the association between birth weight and liver fat is not linear, but rather, a J-shaped relationship with a range of birth weights having a positive association with liver fat.

We further posit that birth weight might not be the ideal proxy for intrauterine nutrition in some populations, especially in mothers who are “constitutionally small”. In support, lean, young males (age 18–22 years) from rural India with low birth weight had similar muscle and liver fat (measured by the radiologic gold standard, magnetic resonance spectroscopy) to those with normal birth weight^28^. Additionally, data on non-nutritional factors that might contribute to low birth weight (maternal smoking and alcohol intake during pregnancy) were not available in this study and might influence its outcomes.

### Strengths and limitations

This is the first report of a study investigating liver fat in a cohort of adults who experienced severe acute malnutrition in early childhood. The study utilized a well-characterized cohort and the use of CT scans allowed for objective, reproducible, quantitative data, compared to the use of ultrasonography which provides only qualitative data which may be subjective. Also, the use of larger ROIs yielded more accurate results and attenuation readings were standardized by using an external phantom to control for scan penetrance. As malnutrition survivors who had abdominal CT scans were similar in age, sex and BMI to those that did not undergo CT scans, the potential for attrition bias was minimal.

We cannot completely exclude a possible selection bias in choosing the 92 study participants. The lack of data on physical activity and current diet is a limitation, although we posit that most of these SAM survivors remained in a non-obesogenic environment as they remained lean on average. Maternal size and diet, breastfeeding and catch-up growth are possible confounders. The study was also limited by the use of a relatively young, lean study population and the lack of birth weight data for community participants. Magnetic resonance spectroscopy and magnetic resonance imaging are more sensitive measures of hepatic fat and would provide better estimates in this young lean group of participants than CT scans which are only sensitive when liver fat exceeds 30% by weight. While CT radiation was a limitation, the radiation dose was minimized by using a scout film followed by 2 single CT slices, as multiple CT slices are known to scatter radiation into adjacent slices thus increasing the radiation dose^29^.

## Conclusions

In this proof-of-concept study, we have provided evidence, for the first time, that SAM survivors, particularly those were severely wasted as infants, are at greater risk of fatty liver if they become obese in later life. Birth weight had the effect of partially masking the association between SAM phenotype and adult liver fat. While the difference in liver fat between groups is very small and of uncertain clinical significance, our data highlight the need to monitor SAM survivors beyond the acute episode to mitigate the risk of unfavourable adult outcomes. It would be instructive to further explore these relationships using a more sensitive measure of liver fat in older SAM survivors, particularly those who are obese.

## Supplementary Information

Supplementary Figure 1.
