# Supplementary material for: Liver fat in adult survivors of severe acute malnutrition
Source: Sci Rep. 2022 Mar 7;12:3690. doi: 10.1038/s41598-022-07749-5 (PMC8901775; doi:10.1038/s41598-022-07749-5)
Supplement: Supplementary file 1 — Supplementary Figure 1. [file 41598_2022_7749_MOESM1_ESM.pdf]

Supplementary Figure: Flow chart detailing recruitment of adult survivors of SAM (n = 92) and community participants (n = 87).

Supplementary Figure 1

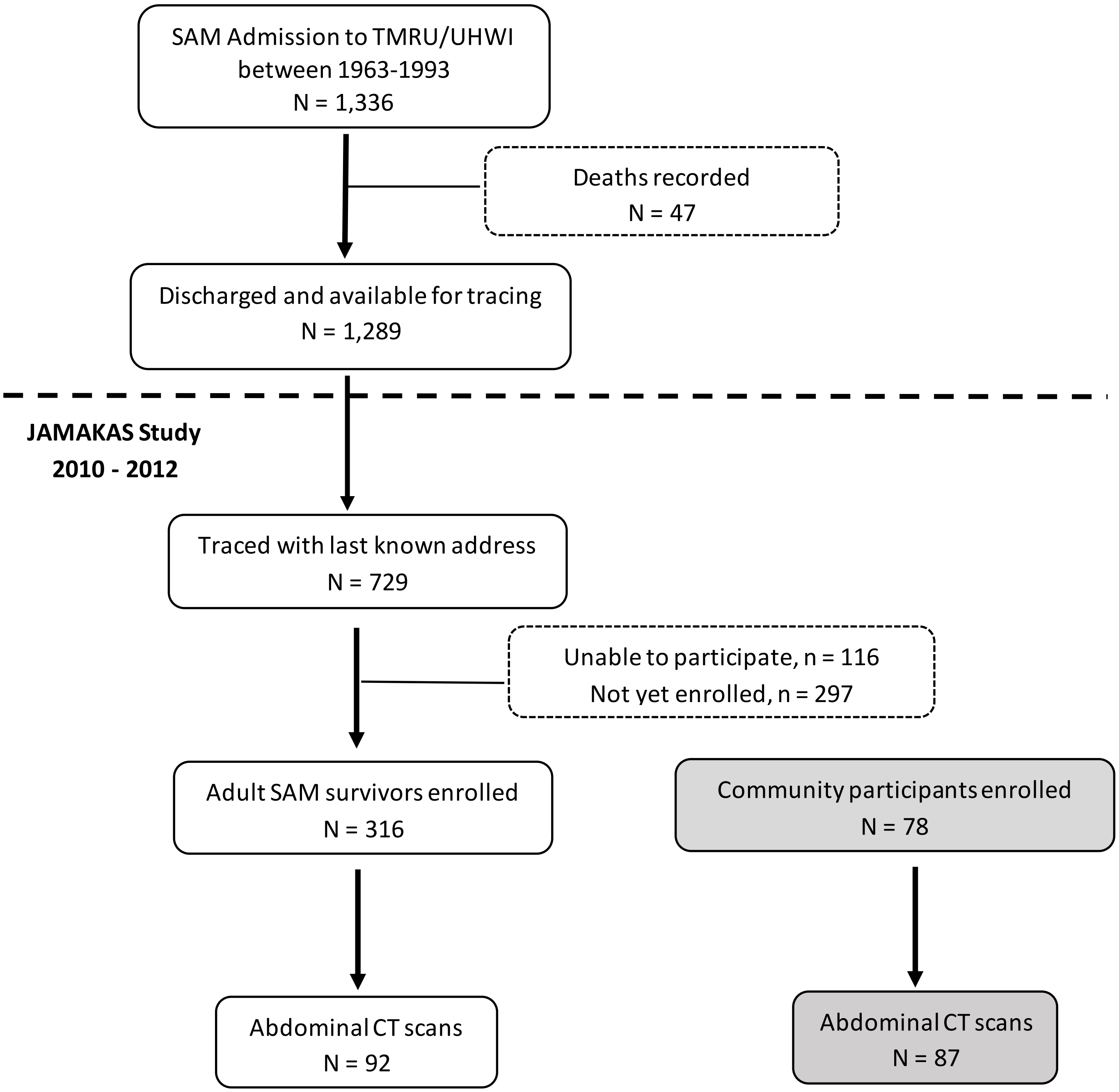

Supplementary Figure 1: Flow chart detailing recruitment of adult survivors of SAM (*n* = 92) and community participants (*n* = 87). "Unable to participate" includes adult survivors of SAM who were unavailable because of migration (*n* = 53), illness (*n* = 19), refusal (*n* = 14), or pregnancy (*n* = 30). TMRU, Tropical Metabolism Research Unit; UHWI, University Hospital of the West Indies; JAMAKAS, Jamaica Marasmus and Kwashiorkor Adult Survivors.
